# Kidney Androgen-Regulated Protein (KAP) Transgenic Mice Are Protected Against High-Fat Diet Induced Metabolic Syndrome

**DOI:** 10.1038/s41598-017-16487-y

**Published:** 2017-11-23

**Authors:** Beatriz Bardaji de Quixano, Josep A. Villena, Miguel Aranda, Gemma Brils, Antoni Cuevas, Théana Hespel, Haizea Lekuona, Cristina Súarez, Olga Tornavaca, Anna Meseguer

**Affiliations:** 1 Fisiopatologia Renal. Centre d’Investigacions en Bioquímica i Biologia Molecular (CIBBIM). Institut de Recerca Vall d’Hebron (VHIR), Barcelona, Spain; 2 Laboratory of Metabolism and Obesity. Vall d’Hebron Institut de Recerca (VHIR), Barcelona, Spain; 3 Departament de Bioquímica i Biologia Molecular. Unitat de Bioquímica de Medicina. Universitat Autònoma de Barcelona, Bellaterra (Barcelona), Spain; 4 Instituto Reina Sofía de Investigación Nefrológica. Fundación Renal Íñigo Álvarez de Toledo, Álvarez de Toledo, Spain; 5 Red de Investigación Renal (REDINREN), Instituto Carlos III-FEDER, Madrid, Spain

## Abstract

Metabolic Syndrome (MS) is reaching epidemic proportions with significant social and economical burden worldwide. Since the molecular basis of MS remains poorly defined, we investigated the impact of KAP, a kidney specific androgen-regulated gene, in the development of high fat-diet (hfd)-induced MS. Tg mice overexpressing KAP specifically in proximal tubule cells of the kidney exhibited reduced body weight and lower liver and adipose tissue weight compared to control littermates when fed a hfd. KAP Tg mice showed diminished adipocyte hypertrophy and reduced hepatic steatosis, significantly correlating with expression of relevant molecular markers and lower lipid content in liver. KAP transgenic were protected from hfd-induced insulin resistance, increased blood pressure and exhibited lower IL-6 serum levels and diminished expression of inflammatory markers in the adipose. Moreover, KAP was localized in the secretory pathway of proximal tubule cells and it is released to the extracellular media, preventing IL-6 induction and STAT-3 activation upon TNFα stimulation. We conclude that KAP, which might act as a hormone-like product in extra-renal tissues, protects Tg mice against hfd-induced MS by preventing inflammatory related events that are mediated, in part, through the IL-6 pathway.

## Introduction

The metabolic syndrome (MS) is defined by a constellation of interconnected physiological, biochemical, clinical, and metabolic factors that directly increase the risk of atherosclerotic cardiovascular disease (ASCVD) and type 2 diabetes mellitus (T2DM)^1^. MS affects over 20% of adults in Western populations and is reaching an epidemic proportion globally^2^.

One of the main underlying risk factors for MS is obesity^3^. Accumulating evidence suggests that chronic inflammation in adipose tissue is instrumental in the development of obesity-related metabolic dysfunction^4,5^. The resulting hyperinsulinemia and hyperglycemia, as well as the release of adipocyte cytokines, play a critical role in vascular endothelial dysfunction, abnormal lipid profile, arterial hypertension (AHT), and vascular inflammation, which work in concert to enhance atherosclerosis^6^. Other obesity-related disorders associated with MS such as fatty liver disease, hyperuricemia and chronic kidney disease (CKD), defined as a glomerular filtration rate (GFR) below 60 ml/min per 1.73 m^2^, are major causes of morbidity and mortality.

Recent epidemiologic studies have demonstrated that patients with MS are also at increased risk of microalbuminuria, an early marker of glomerular injury, endothelial dysfunction and/or CKD. Data from the NHANES III database, which includes more than 6000 adults, revealed that the multivariate-adjusted risk for both microalbuminuria and CKD was significantly raised in individuals with MS, and that this risk increased progressively with the number of the syndrome’s components detected in each patient^7^.

How the functional changes in the kidney affect MS pathophysiology remains speculative. Factors such as insulin resistance (IR), inflammation, renal endothelial dysfunction, oxidative stress, altered renal hemodynamics, activation of the renin-angiotensin-aldosterone system (RAAS) and the sympathetic nervous system (SNS)^8^ may play a role.

Kidney-androgen regulated protein, which is one of the most abundant and specific genes expressed in proximal tubule epithelial cells^9^, undergoes a strict and unique regulation by thyroid and sexual steroid hormones, mainly androgens, in the proximal tubules^10^. The function of KAP, which shares no significant homology with other known proteins in various databases, is almost unknown. Our results in mice with transgenic overexpression of KAP in the proximal tubule demonstrated for the first time a key role for this protein in the induction and or activation of several extra renal pathways, including cardiovascular disease and arterial hypertension^11,12^. It was noteworthy that KAP provided a mechanistic insight into the higher prevalence of arterial hypertension in males.

In this study, we describe the impact of KAP overexpression in the kidney proximal tubule on the development of metabolic syndrome induced by high fat diet.

## Results

### KAP overexpression improves diet-induced obesity

**Figure 1:**
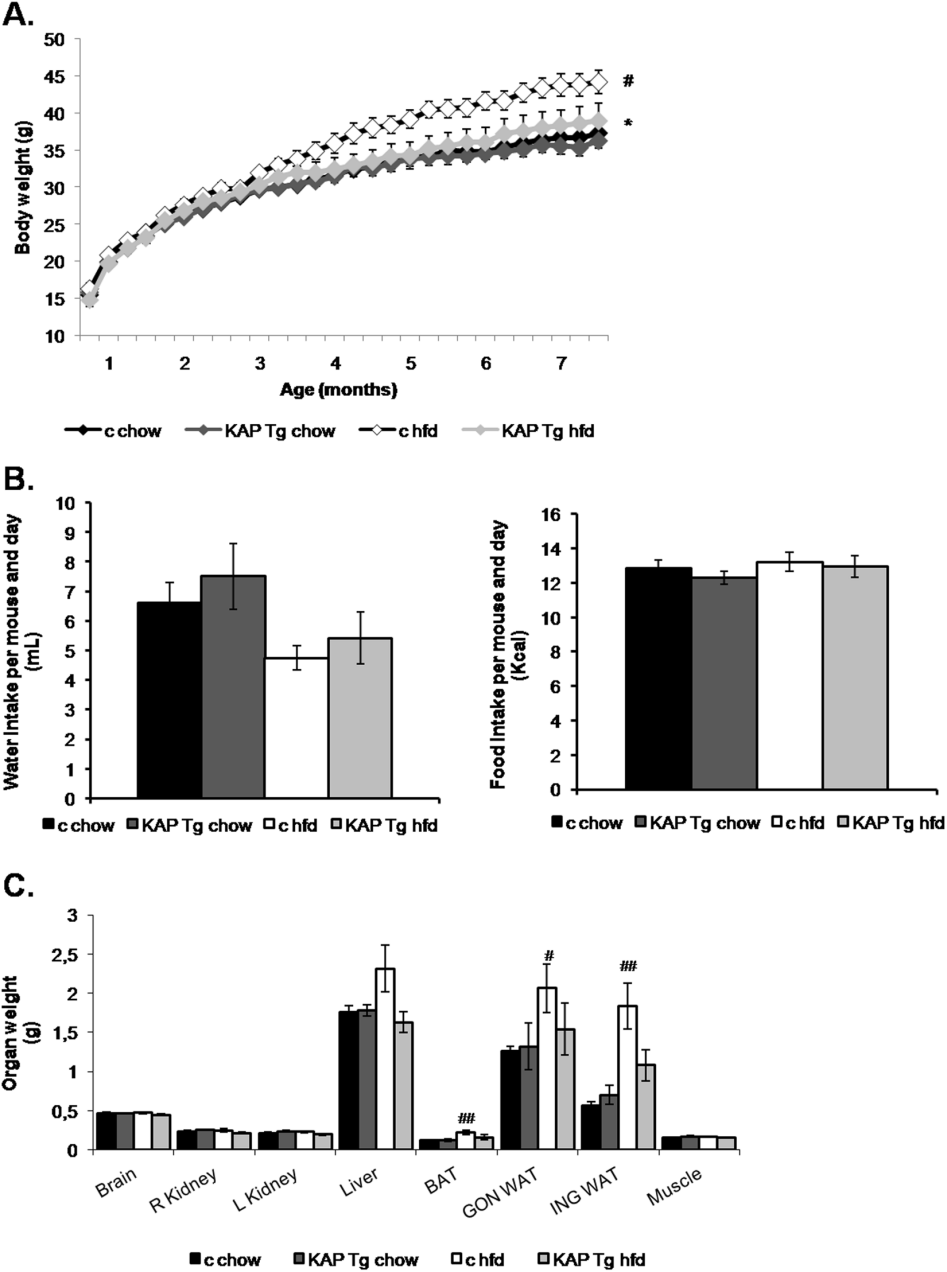
Body weight control. (**A**) Weekly body weight of KAP Tg male mice (Tg) and control male littermates (c) fed on regular control diet (chow) or high fat diet (HFD). Results correspond to mean values from 3 independent set of experiments including n = 8 animals/group. Statistical analysis performed on area under the curve (AUC). *p < 0.05 between controls and Tg. #p < 0.05 between chow and HFD. (**B**) Weekly water (in grams) and food (kcal) intake. Results for this figure correspond to mean values from 1 experiment including control and Tg mice fed on regular chow or HFD (n = 8 per group). (**C**) Organ’s weight at the time of sacrifice at 7 months of life. Results correspond to mean values from 2 independent set of experiments including n = 8 animals/group. Significant differences between chow and HFD, ##p < 0.01. Error bars correspond to SEM.

**Figure 2:**
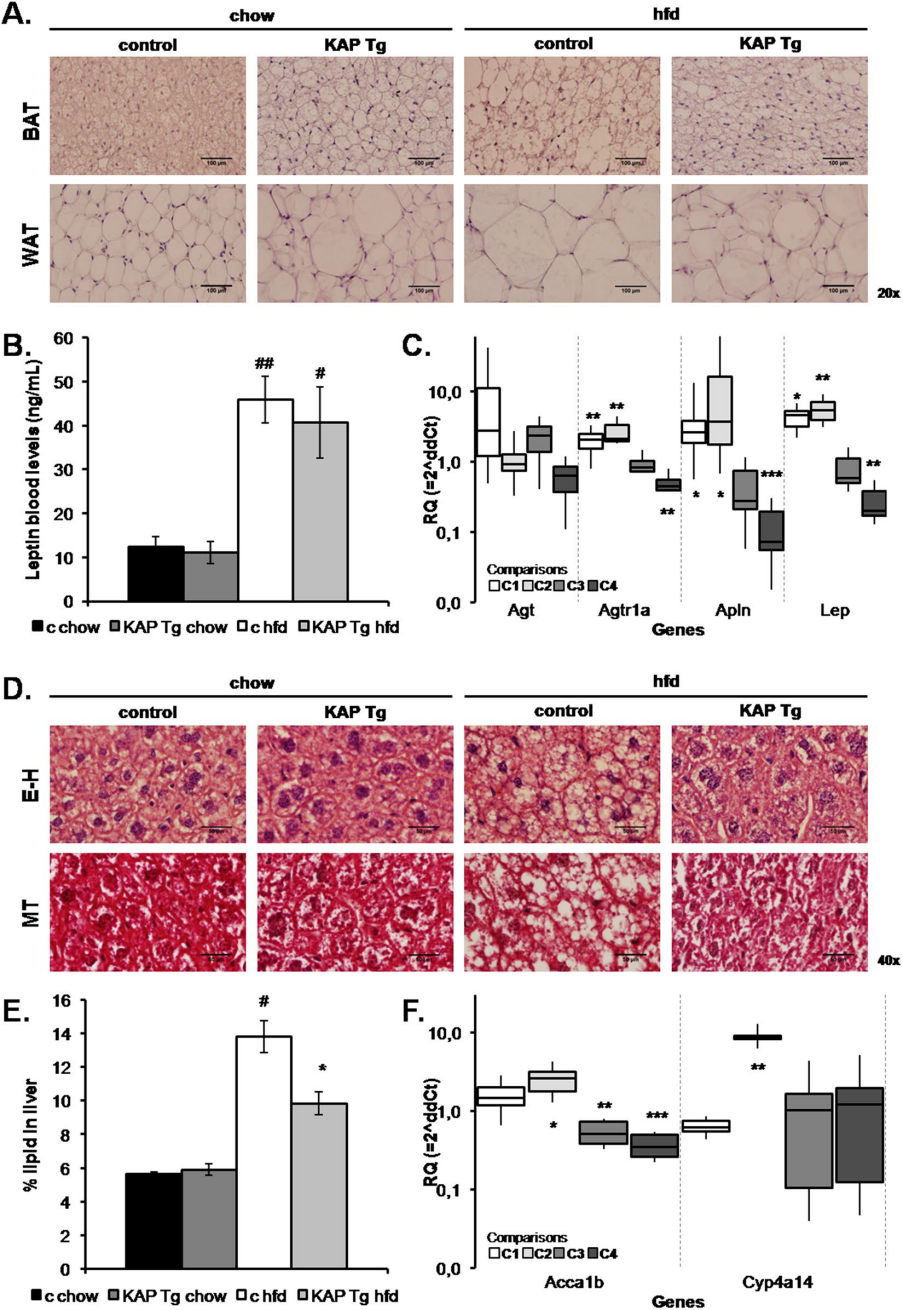
Adipose tissue and liver analyses. (**A**) Eosin-haematoxylin (**E**–**H**) staining of white and brown adipose tissues. (**B**) Serum leptin levels measured in 6-month old 5-h fasted mice. Results correspond to mean values from 2 independent set of experiments including n = 8 animals per group. #p < 0.05, ##p < 0.05, between chow versus HFD. Error bars correspond to SEM. (**C**) Comparative differential gene expression assays in white adipose tissues were performed by qRT-PCR using microfluidic cards. The figure represents the relative quantification (RQ) of gene expression differences between 4 experimental groups, with two variables under study including the Tg vs control condition and the hfd vs regular chow diet. Data is expressed in a logarithmic scale meaning that values above or below 1 correspond to up- or down-regulated expression, respectively. The box-plots represent the median value and the standard deviation (SD) for each comparison: C1, C2, C3, C4. The vertical dashed lines limit the information for each one of the four genes selected. C1 = Comparison 1 “Tg chow - c chow”, represents the differences of gene expression in relation to the genotype, calculated by the Livak method; C2 = “c HFD - c chow” represents the differences of gene expression in relation to the diet; C3 = “Tg HFD – c HFD” represents the impact of the genotype on HFD effects; C4 = “(Tg HFD – c HFD) – (tg chow- c chow)” represents the effects that HFD produces in Tg versus control littermates once eliminated the effects caused by the Tg condition itself. *p < 0.05; **p < 0.02; ***p < 0.001. (**D**) Eosin-haematoxylin (E-H) (upper panel) and Masson trichrome (MT) staining (lower panel) of livers from control and Tg mice fed with chow or high fat diet. (**E**) Relative hepatic lipid content. Results for this figure correspond to percentage of lipid weight over total liver weight from a single experiment (n = 8 per group). *p < 0.05 control vs Tg, #p < 0.05 chow vs HFD. Error bars correspond to SEM. (**F**) Comparative differential gene expression assays in liver tissues were performed by qRT-PCR using microfluidic cards. The groups, according to the genotype and the diet, were compared as described in panel C. *p < 0.05; **p < 0.02*.

KAP Tg and control littermates (WT) were fed either a regular diet (chow) or hfd to induce MS. In spite of similar water and food intake (Fig. 1B), hfd-fed KAP overexpressing mice exhibited reduced body weight (Fig. 1A), as well as, lower liver and adipose tissue weight than controls (Fig. 1C). Consistently with reduced fat mass, hfd-fed Tg mice showed lower lipid accumulation in white and brown adipocytes than controls (Fig. 2A). In hfd-fed WT mice, adipose hypertrophy and augmented adipocyte size correlated with leptin circulating levels (Fig. 2B) and up-regulation of leptin mRNA in the adipose (Fig. 2C). Leptin mRNA was strongly down-regulated in hfd-fed Tg. These results paralleled those found for the *Apelin* gene (Fig. 2C), which encodes for a peptide hormone linked to IR and obesity^13^. Up-regulated adipose RAS in obese individuals, contributes to IR and hypertension. We found that angiotensinogen (Agt) and receptor of Angiotensin II type 1 (AT1) are up-regulated in adipose of Tg and that AT1 is also up-regulated in hfd fed controls. Once again, up-regulation of AT1 by hfd in WT mice was significantly blunted in Tg (Fig. 2C). Figure 2C represents the relative quantification (RQ) of gene expression differences between the four experimental groups, with two variables under study including the Tg vs control condition and the hfd vs regular chow diet. It shows that changes in gene expression produced by the Tg (C1) or the hfd (C2) situations are lost when both conditions occur simultaneously (C3). (C4) indicates that the effects of the HFD in the Tg are actually stronger than those shown in C3, since the C3 comparison includes the effects of the Tg condition itself, observed in the chow diet-fed Tg (C1).

Liver histological analyses confirmed hepatic lipid accumulation in hfd-fed control mice but not in Tg (Fig. 2D,E), indicating that KAP overexpression protects from hfd-induced hepatic steatosis. The most relevant mRNA changes in liver were the significant up-regulation of Cyp4A14 and Acaa1b genes in hfd-fed controls. Acaa1b became significantly down-regulated in hfd-fed Tg when compared to controls; thereby, reverting the phenotype induced by the diet (Fig. 2F).

### KAP transgenic mice exhibit improved glucose homeostasis upon a hfd feeding

**Figure 3:**
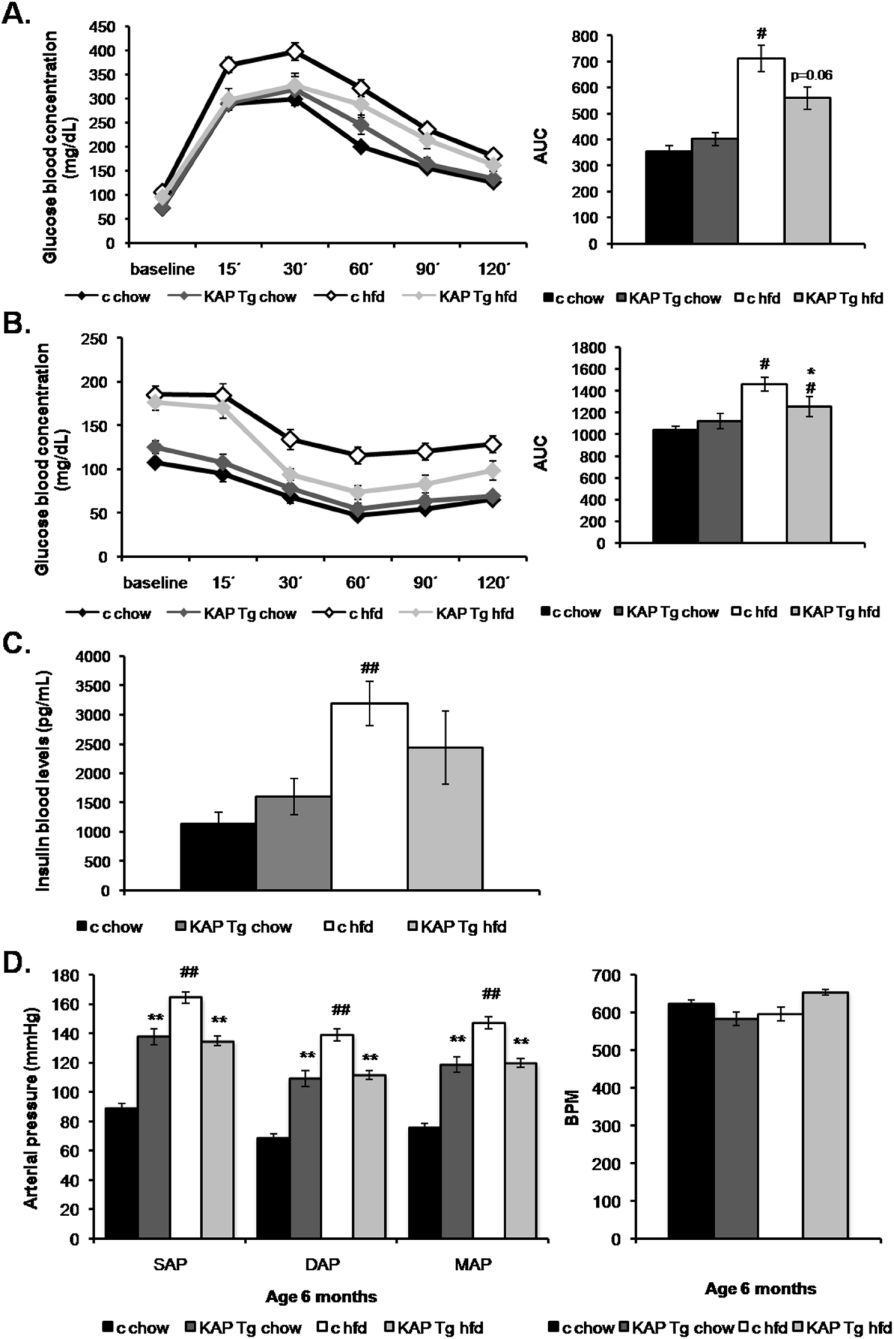
Glucose metabolism and Hemodynamic analyses. Glucose metabolism assessment in Tg mice and control littermates fed on regular control diet or high fat diet, at 6 months of age. Results from three independent experiments, (n = 8 per group) are shown. (**A**) Glucose tolerance test (GTT) in awaken animals after 12 h o/n fasting. Results correspond to mean absolute values in basal conditions (pre glucose bolus) and at 15, 30, 60, 90 and 120 min after glucose bolus. Statistical analysis performed on area under the curve (AUC). ##p < 0.05 chow versus HFD. (**B**) Insulin tolerance test (ITT) in awaken animals after 5 h o/n fasting. Mean absolute values in same conditions as in A. Statistical analysis on AUC *p < 0.05 control vs Tg, #p < 0.05 chow vs HFD. (**C**) Insulin levels measured in 6-month old 5-h fasted mice, using the Mouse Serum Adipokine Milliplex kit. Two independent set of experiments (n = 8 per group). Blood samples were taken after 5-h over fasting. *p < 0.05 chow vs HFD. (**D**) Morning tail-cuff measurements of systolic (SAP) diastolic (DAP) and mean arterial pressure (MAP) in Tg and control littermate mice fed on standard control diet (chow) or high fat diet (HFD), at 6-month of age (left panel). Mean and SEM from one experiment (n = 8 per group) are represented. Statistical analysis on AUC **p < 0.01 control vs Tg, ##p < 0.01 chow vs HFD. Heart rate measurements in the four groups of mice (right panel). For all panels error bars correspond to SEM.

Glucose tolerance test showed no significant differences between chow-fed WT and Tg mice. Hfd induced a severe glucose intolerance in WT whereas KAP overexpressing mice were partially protected (Fig. 3A). Similarly, insulin tolerance tests revealed that KAP protected Tg mice from the development of IR when fed a diabetogenic fat rich diet (Fig. 3B). Insulin levels, in serum of 5h-fasted animals, revealed that the hfd-induced compensatory hyperinsulinemia in WT mice, was significantly prevented in Tg (Fig. 3C), supporting the notion that KAP protects from hfd-induced IR.

Hfd-induced obesity is often associated with alterations in blood lipid profile. No major differences in serum FFA o triglycerides were observed between Tg and WT, either fed with chow or hfd. However, KAP Tg mice showed lower total cholesterol levels than WT littermates when fed with hfd (Table 1), indicating that KAP protects from the hypercholesterolemia associated to hfd-induced obesity.

**Table 1:** Clinical chemistry parameters.

		chow		hfd	
		control	KAP Tg	control	KAP Tg
Glucose	Mean ± sem (mg/dl)	107,81 ± 4,25	125,50 ± 7,34 (*)	185,31 ± 9,62 (##)	176,94 ± 9,49 (##)
Total cholesterol	Mean ± sem (mg/dl)	139,02 ± 4,83	128,37 ± 6,81 (n.s.)	226,06 ± 9,98 (##)	155,84 ± 6,41 (**)(##)
Triglicerides	Mean ± sem (mg/dl)	55,26 ± 2,95	51,36 ± 5,38 (n.s.)	56,36 ± 5,00 (n.s.)	62,55 ± 4,48 (n.s.)
FFA	Mean ± sem (mM)	0,51 ± 0,02	0,48 ± 0,04 (n.s.)	0,55 ± 0,03 (n.s.)	0,69 ± 0,03 (**)(##)

Six-month old animals were fasted for 5 h. Serum glucose levels correspond to mean values from three independent experiments of controls and transgenic mice fed on chow or HFD (n = 8 per group). *P < 0.05 control vs Tg, ^##^P < 0.01 chow vs Tg. Serum total cholesterol, triglycerides and FFA quantification correspond to mean values from two independent experiments, **P < 0.01 control vs Tg, ^##^P < 0.01 chow vs Tg.

### Arterial blood pressure in Tg and control mice on chow or hfd

Consistent with the concept that obesity increases the risk of developing hypertension^14^, hfd-fed WT mice exhibited a significantly higher systolic, diastolic and mean arterial pressure (SAP, DAP, and MAP, respectively) than chow-fed WT mice (Fig. 3D, left panel). Albeit the characteristic hypertensive phenotype of KAP Tg mice^11,12^, hfd-fed Tg mice maintained the same blood pressure than those fed with chow (Fig. 3D, left panel), indicating that KAP overexpressing mice are resistant to the hypertensive effects prompted by hfd. Differences in blood pressure do not correlate with alterations in heart rate (Fig. 3D, right panel). SAP and DAP measurements, evaluated during morning and afternoon periods gave similar results (Fig. S1).

### Serum and tissue inflammatory markers in WT and Tg mice

**Figure 4:**
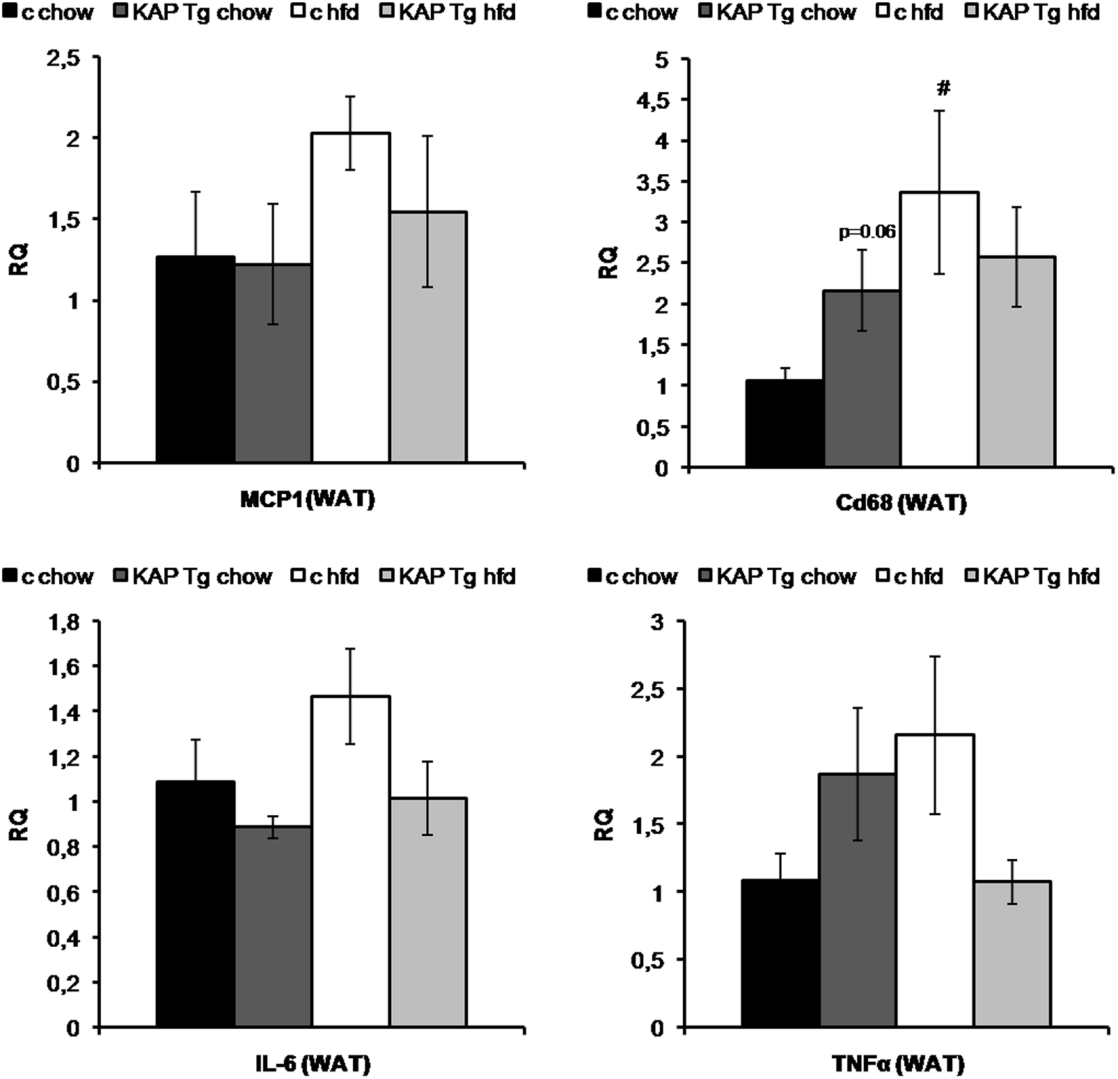
Gene expression assays. Quantitative RT-PCR gene expression assays (qReal Time TaqMan probes PCR) in white adipose tissues of Tg and control littermate mice fed with chow or HFD, upon sacrifice at 7-month of age. Relative expression of MCP-1, Cd68, IL-6 and TNF-α pro-inflammatory genes are given in fold change (FC) 2^−ΔΔCt^. Peptidyl-prolyl isomerase A (CypA) probe was used as endogenous control (n = 8 per group), #p < 0.05 chow vs HFD. Results correspond to mean values from three independent set of experiments including n = 8 animals/group. For all panels error bars correspond to SEM.

Since MS represents a state of chronic low-grade inflammation, we aimed to observe circulating levels of inflammatory markers (i.e. IL-6, PAI-1, TNF-α and resistin) in sera from WT and Tg mice fed with chow or hfd. Resistin levels appeared significantly up-regulated in Tg and in hfd-fed WT mice, while PAI-1 followed the opposite pattern (Table 2). Correlating with the development of obesity and IR, IL-6 serum levels showed an increment in hfd-fed WT mice that was blunted in Tg mice, indicating that KAP prevents systemic hfd-induced up-regulation of IL-6, with no effect on resistin and PAI-1 levels (Table 2). In this same assay we intended to measure TNF-α levels but it did not work under our conditions of assay. It is described that serum TNF-α levels are particularly susceptible to changes in sample collection and storage procedures which might preclude the obtention of a measurable value. The effects of hfd on the expression of inflammatory markers in WAT, assessed by qRT-PCR, revealed that MCP-1, TNF-alpha and IL-6 mRNA levels were increased in WT mice by hfd, in correlation with a statistically significantly up-regulation of mRNA levels for the macrophage marker CD68 (Fig. 4). Interestingly, these inflammatory markers remain lower in hfd-fed Tg mice, indicating that KAP overexpression might counteract the inflammatory impact of hfd in adipose tissue.

**Table 2:** Cytokines and adipokines measurement.

		chow		hfd	
		control	KAP Tg	control	KAP Tg
IL-6	Mean ± sem (pg/dl)	30,837 ± 3,453	32,978 ± 3,345 (n.s.)	45,765 ± 8,192 (n.s.)	33,608 ± 3,290 (n.s.)
PAI-1 (total)	Mean ± sem (pg/dl)	8247,600 ± 2078,861	10581,000 ± 3179,562 (n.s.)	6732,208 ± 1212,884 (n.s.)	6943,542 ± 1654,839 (n.s.)
Resistin	Mean ± sem (pg/dl)	5725,100 ± 530,788	5254,227 ± 378,057 (n.s.)	7483,042 ± 294,662 (#)	7883,607 ± 754,882 (p < 0,06)

IL-6, PAI-1 (total) and resistin levels in 6-month old animals using the Mouse Serum Adipokine Milliplex kit. Blood samples were taken after 5-h over morning fasting. Results correspond to mean values from 2 independent set of experiments including n = 8 animals per group, ^#^p < 0.05 chow vs HFD.

### KAP over expression prevents TNFα-mediated IL-6 transcription in the proximal tubule derived cell line HK-2

**Figure 5:**
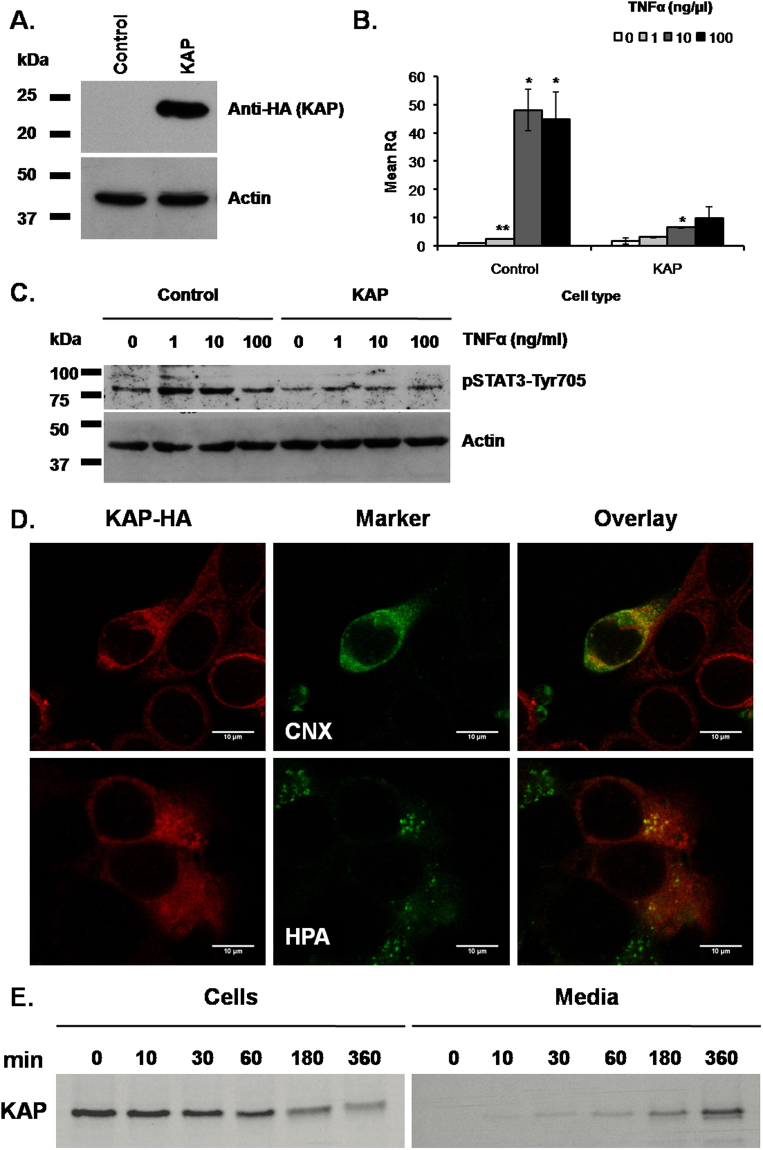
KAP effects on IL-6 expression by TNF-α. KAP subcellular location and secretion. (**A**) KAP-HA expression (KAP) in the HK-2 cell line. Empty lentiviral vector transduced cells were used as a control (Control). KAP-HA expression by determined by Western blot analyses using anti-HA antibody (ROCHE). (**B**) IL-6 mRNA induction upon TNF-α stimulation in HK-2 cells. Control and KAP transduced HK-2 cells were treated with increasing doses of TNF-α (0,1,10 and 100 ng/μl) for 24 h and IL-6 expression performed by qRT-PCR using IL-6 TaqMan probes. CypA was used as endogen control. Results represented in this figure are the average of three independent biological replicas. *p < 0.05; **p < 0.01, all versus TNF-α at 0 ng/μl, one-way ANOVA. Error bars correspond to SEM. (**C**) Effects of KAP-HA expression (KAP) on p705YSTAT-3 levels in HK-2 cells treated with increasing doses of TNF-α (0,1,10 and 100 ng/μl) for 24 h, in comparison to control transduced cells. Actin and total STAT-3 proteins were following the same expression pattern in all samples (not shown). The experiment represented in Fig. 5C has been done once. Samples derive from the same experiment and that gels/blots were processed in parallel. (**D**) Mouse proximal tubule derived PCT3 cells transiently transfected with KAP were incubated with anti-HA antibody (Roche) (upper and lower left panel). For subcellular co-localization cells were co-incubated with antibodies anti Calnexin for endoplasmic reticulum detection (upper middle panel), or with the Helix pomatia (HPA) Alexa Fluor 488 (lower middle panel) for Golgi detection. Overlay images indicate that KAP co-locates with endoplasmic reticulum (ER) and Golgi specific markers (upper and lower right panels, respectively). Results in Fig. 5D have been repeated at least three times using different biological replicas. (**E**) For metabolic labelling and pulse-chase experiments, pHA-CMV/KAP-HA transiently transfected PCT3 cells were incubated with Methionine and Cysteine depleted labelling media and further incubated with Trans 35^S^-Label. Cells and conditioned media were harvested at different times. S^35^-labeled KAP recombinant protein was immunoprecipitated from crude cell extracts (left panel. 1 o/n exposition) and conditioned media (right panel. 5 o/n exposition) with anti-HA antibodies. IP- proteins were run in SDS-PAGE and exposed to auto-radiographic films. Levels of KAP protein in crude cell extracts were diminishing over time in good correlation with increments found in conditioned media. Western blot images shown in this figure have not been cropped and each come from a single representative gel/blot experiment.

Because KAP overexpressing mice do not exhibit ectopic KAP mRNA expression and this is confined to proximal tubule cells^11^, we next aimed to determine whether the effects of KAP on IL-6 levels in hfd-fed Tg would directly relate with KAP or, rather, with other systemic events occurring *in vivo*. The effects were investigated in the HK-2 cell line, in basal conditions and upon TNFα stimulation. Stably transduced HK-2 cells, carrying control or KAP-HA lentiviral vectors, were treated with increasing doses of TNFα, and IL-6 mRNA levels measured by qRT-PCR. Figure 5A shows the successful overexpression of exogenous KAP-HA fused protein in HK-2 cells and the stimulatory effects of TNFα on IL-6 mRNA levels (Fig. 5B), which were significantly prevented in the presence of KAP (Fig. 5B, right). Correlating with diminished IL-6 expression, p705YSTAT-3 levels in TNFα stimulated cells were also lowered in the presence of KAP, suggesting the functional impact of KAP on the IL-6/gp130/STAT-3 signaling pathway in proximal tubule cells (Fig. 5C). Non-significant changes on cell viability were observed as a consequence of KAP overexpression or TNFα treatments (not shown).

Our results suggest that KAP overexpression in a pro-inflammatory context, as the one produced by hfd, reduces inflammation and prevents the pathological signs of MS induced by hfd.

### The KAP protein is located in the secretory pathway and is released to the extracellular media of cultured proximal tubule cells

To decipher how KAP, which is exclusively expressed in kidney, could exert metabolic effects in distal tissues, including the liver and the adipose, we aimed to detect the KAP protein in plasma samples of Tg and control mice by WB assays. We did not get any specific signal perhaps due to low circulating KAP levels or to the poor sensitivity of our antibody. Commercial ELISA kits proved to be non-specific for the KAP protein and therefore not suitable to detect and measure KAP in plasma samples. Then, we aimed to observe KAP subcellular location in transiently transfected mouse proximal tubule derived PCT3 cells. By indirect immunofluorescence and confocal microscopy in KAP-HA transfected PCT3 cells, we observed that KAP overlays with endoplasmic reticulum (ER) and Golgi apparatus specific markers, indicating that KAP is located in the secretory pathway (Fig. 5D upper and lower panels, respectively). Pulse-chase experiments using S^35^-methionine/cysteine metabolic labeling in KAP-HA transfected PCT3 cells showed a strong signal for the KAP protein in crude extracts at pulse time zero (Fig. 5E, left panel) that was taken to the secretory pathway with time. KAP was detected in the conditioned media after 30 min chase, reaching maximal expression at 6 h (Fig. 5E, right panel). Levels of KAP protein in crude cell extracts were diminishing over time in good correlation with increments found in conditioned media. These *in vitro* experiments proved that KAP is secreted from proximal tubule epithelial cells to the extracellular media and, possibly, into the bloodstream *in vivo* where it might act as a hormone-like product in extra-renal tissues.

## Discussion

Our study provides relevant and novel information on the impact that the kidney can exert on the pathophysiology of the MS. Diminished expression of the KAP gene has been associated to diabetic nephropathy and atherosclerosis^15,16^, reinforcing the concept that KAP might be involved in processes linked to metabolic disorders. Because MS constitutes an extraordinary health problem worldwide, we aimed to investigate the role of KAP overexpression in proximal tubule and, by extension, the contribution of the kidney in MS pathophysiology.

In the present report, we have observed that in WT mice hfd induces a MS phenotype that includes severe hypertension and significant body gain weight associated to increased fat mass, highly hypertrophied adipocytes, impaired glucose homeostasis and IR. Serum levels of leptin, insulin, resistin and IL-6 inflammatory markers were higher in hfd-fed WT mice, correlating with the MS phenotype. Adipocyte hypertrophy resulted in significantly up-regulation of apelin, leptin and AT1 mRNA levels; as well as, augmented expression of inflammatory markers in the adipose including TNF, IL-6 and CCL2/MCP-1, that correlated with augmented macrophage content, as indicated by the significant overexpression of CD68. These results fit with reports describing that RAS over-activation in adipose tissue has a metabolic and inflammatory impact in obesity^17–20^, as well as, an effect on the differentiation of monocytes from hematopoietic progenitors^21^, influencing their chemotaxis, through up-regulation of CCR2/CD68^22^. Blocking RAS with angiotensin-converting enzyme (ACE) inhibitors and AT1 blockers represent an established therapy for hypertension, diabetic nephropathy and arteriosclerosis^23^. The hfd provoked an overt hepatic steatosis in WT mice that correlated with a significant increment of hepatic lipids and augmented mRNA levels of the fatty-acid, steroid and cholesterol biosynthesis related genes Cyp4A14 and Acaa1b, in liver^24,25^. Although the underlying mechanisms linking obesity to hepatic lipid accumulation and IR are incompletely understood, TNFα and IL-6 production in adipose tissue are critical for the development of steatohepatitis and NFκB has been recognized as an obligatory mediator of most of these TNFα responses^26^. Blocking IL-6 cytokine signaling in adipocytes has been recently suggested as a novel approach to blunt detrimental fat-liver crosstalk in obesity^27^.

Up-regulation of IL-6 mRNA in adipose tissue and augmented IL-6 serum levels found in hfd-fed WT mice were blunted in KAP Tg littermates, indicating that KAP beneficial effects preventing hepatic steatosis and IR might be related with its capacity to impair IL-6 production and inactivation of the IL6/gp130 axis in hfd-fed mice. To assess whether disrupted IL-6 production in hfd-fed KAP Tg could be attributed to direct KAP action on IL-6 expression or to other indirect systemic effects occurring in Tg, we used HK-2 cells over-expressing a fused KAP-HA protein, to observe the putative impact of KAP on IL-6 expression, upon treatment with TNFα. Elevated levels of TNFα are detected in the bloodstream and in the peripheral tissues of insulin-resistant mice and, both, TNFα neutralization and deficiency each prevent high-fat diet-induced IR^28^. Our results clearly demonstrated that KAP overexpression in HK-2 cells has a direct effect on IL-6 production by TNFα. Since TNFα induces the NFκB pathway, which has a key role in the development of inflammation associated metabolic diseases^29^, we postulate that KAP positive effects may be linked to the blunting of the NFκB signaling pathway. Moreover, we have shown that KAP overexpression in HK-2 cells has an impact in the IL-6/gp130/JAK/STAT-3 pathway preventing 705YSTAT-3 activation, likely due to the impairment on IL-6 production. This is a very important pathway in obesity since increased circulating leptin levels lead to development of leptin resistance by chronic activation of JAK/STAT3 in the CNS, whereas in the peripheral organs chronic IL-6-induced JAK/STAT3 impairs insulin action^30^. Whether KAP can activate/block these pathways by a direct action in target tissues or through indirect mediators is an open question. Because KAP is located in the secretory pathway and was also found in conditioned media of cultured cells, it is plausible to think that it might act as a kidney-specific hormone-like molecule that binds to putative KAP receptors in distal tissues.

It is intriguing to understand the sometimes opposite metabolic actions of KAP in basal and in hfd conditions. While KAP overexpression in proximal tubule cells counteracts negative effects produced by an obesogenic diet, KAP can also promote hypertension, proteinuria, glucosuria and focal segmental glomerulosclerosis in chow-fed Tg mice. It was reported that those effects are in part due to over-activation of Cyp4A, systemic oxidative stress^11^, sympathetic and renin-angiotensin (RAS) systems^12^. Obesity induced by hfd is characterized by increased circulating levels of the adipocyte-derived hormone leptin, which can increase sympathetic nerve activity and raise blood pressure through up-regulation of central RAS and pro-inflammatory cytokines. Systemic oxidative stress is also part of the alterations reported during chronic obesity^31^ and ROS and the NFκB pathway interact to each other in many ways. Depending on the context, ROS can both activate and inhibit NFκB signaling^32^. Our results suggest that KAP may indirectly activate the NFκB pathway through ROS production and possibly behave as a putative agonist of IKbα, in the presence of other pro-inflammatory stimuli. KAP and IKbα share the same serine/threonine residues of a PEST domain that permits NFκB activation by ROS^33^. This H_2_O_2_-inducible phosphorylation of IKbα does not depend on I kappa B kinase (IKKB) activation, but involves CK2. Upon phosphorylation by CK2, IKbα and KAP are degraded by calpain proteases or through the proteasome^33,34^. Aligned with this rationale, we had earlier described that, *in vitro* ^35^ and also in Tg mice^34^, KAP overexpression protects from cyclosporine A-induced proximal tubule injury. As recently reported, inflammation represents a key pathogenic event leading to kidney damage and fibrosis in cyclosporine nephrotoxicity^36^.

Overall, our results indicate that under regular diet feeding, KAP overexpression activates pathways that are also activated by hfd in wild type mice. When the Tg and the hfd conditions are associated, KAP might saturate key enzymes and/or receptors in those common pathways, functionally counteracting the effects of hfd in MS development. Because KAP is secreted to conditioned media, we claim that it might act as a hormone-like product in extra-renal tissues, constituting a potential target for therapeutic interventions against MS.

## Experimental Procedures

### Animals

The generation of KAP Tg mice has been described^11^. Tg and control littermate male mice were fed ad libitum with standard chow diet (2018 Harlan Global Diet 18% Protein Rodent Diet) or with high fat diet (TD.06435 Tekland custom research diet: 45% kcal fat, 35% kcal carbohydrates, 20% kcal protein) from weaning until euthanasia, for a 6-months period. Three independent set of experiments were performed along time (n = 8/group/experiment).

Mice were housed under constant conditions of temperature (22 °C) and humidity (60%) and subjected to a 12-h light/dark cycle. Mouse body weight was measured weekly throughout the duration of the experiment. Mice were euthanized under isoflourane anesthesia and organs collected for further analyses. Organs were weight and kept frozen at −80 °C or fixed with formalin 4% for further studies.

All experimental procedures were in compliance with the rules of the European Union guide for the Care and Use of Laboratory Animals. All procedures were approved by the Animal Experimentation and Ethics Committee of the Vall d’Hebron Research Institute (CEEA 07/11).

### Blood pressure measurements

Arterial pressure was measured using tail-cuff method (Niprem 546, Cibertec SA, Madrid, Spain) adapted for mice as previously described^11,12^.

### Glucose and insulin tolerance tests

For glucose tolerance tests, mice were given an intra-peritoneal injection of glucose (1 g/Kg) after 12 h over-night fasting. For insulin tolerance tests, mice were first fasted for 5 h and then injected with insulin (0.75 IU/Kg). Blood glucose concentration was determined with an ELITE glucometer (Bayer).

### Serological parameters

Blood from KAP Tg mice and control littermates was collected after a 5 h fasting. Concentration of Triglycerides and total cholesterol in serum was determined using commercial kits based on the Trinder colorimetric method (FAR Diagnostics, Italy)^37^. Free fatty acids content was analyzed with the NEFA-C kit (Wako Chemicals GmbH, Germany). Insulin, leptin, resistin, IL-6 and PAI-1 were determined with the Milliplex Map Mouse Serum Adipokine Panel multiplex assay (Merck Millipore).

### Hepatic lipid content

Neutral Lipid fraction was isolated from mouse livers by the Folch method in order to quantify the lipids content^38^.

### Histological analysis

Paraffin-embedded liver, white and brown adipose tissue sections were stained with hematoxylin and eosin and Masson’s trichrome, according to standard procedures.

### RNA isolation and cDNA synthesis

Total RNA was isolated from tissues with Trizol (Life Technologies) following the manufacturer’s instructions. Total RNA was retro-transcribed using the High Capacity RNA-to-cDNA Master Mix (Applied Biosystems) and used to perform gene expression analyses by qRT-PCR using TaqMan® Gene Expression Master Mix (Applied Biosystems) or microfluidic cards.

### Microfluidic cards

Microfluidic cards (ref.: Applied Biosystems Taqman® Low Density Array, format 32, Part. N 434799 G) were used to evaluate differential expression of genes involved in RAAS axis, oxidative stress, inflammation and glucose and lipid metabolism in white adipose tissue and liver. Samples were randomized among the cards in order to ensure that no samples from the same experimental group were loaded in the same card or in the same position of a different card. The best endogenous genes were sought among those included commercially in the cards using DataAssist ™ software v. 2.0, so that data were corrected using the geometric mean of the more stable endogenous genes (GAPDHs, CypA and β-actin).

### Real-time qRT-PCR

qRT-PCR for IL-6, TNF-α, MCP-1 and CD68 was performed in the 7500 Real-Time PCR System (Applied Biosystems) using TaqMan probes. Peptidyl-prolyl-isomerase A (CypA) probe was used as reference gene.

### KAP expression in mouse proximal tubule cells

The pHA-CMV/KAP-HA mammalian expression vector containing the full coding sequence of the mouse *Kap* gene fused to the HA epitope has been previously reported^34^. For transfection experiments, PCT3 cells were grown to about 50% confluence in medium containing 2% fetal calf serum (FCS). Transfections were done in serum-free medium with the indicated cDNA using 0.4–0.8 μg/well of a 24-well plates or 5 μg/ 6 cm dish by LipofectAMINE PLUS (Life Technologies) method according to the supplier’s manual and as previously reported^10,34^.

### Immunocytochemistry in cultured cells

Transfected PCT3 cells were fixed in −20 °C methanol for 4 min at room temperature. After blocking for 30 min in blocking buffer (1% bovine serum albumin in PBS), cells were incubated for 1 h at room temperature with anti-HA (1ug/ml, Roche Molecular Biochemicals) diluted in blocking buffer. For subcellular co-localization analyses cells were co-incubated with antibodies anti Calnexin (1:50 Stressgen), which detects the endoplasmic reticulum, or with the Helix pomatia (HPA) Alexa Fluor 488 (Molecular Probes) that binds to the Golgi Apparatus. The cells were washed in PBS and further incubated with FITC-conjugated anti-rat IgG (1:350, Sta. Cruz Biotechnology) or TRITC-conjugated anti-rabbit antibodies (1:300, Sigma). After washing with PBS, coverslips were mounted on a glass slide and images were obtained using a Leica DM IRBE confocal microscope.

### Metabolic labelling. Pulse-chase experiments

pHA-CMV/KAP-HA transiently transfected PCT3 cells were incubated with Methionine and Cysteine depleted labelling media for 30 min, and further incubated with 0,1 mCi/ml Trans 35^S^-Label (ICN PHARMACEUTICALS, INC.) for 30 min at 37 °C and 5% CO2. Upon washing, cultures were incubated with complete media and cells and conditioned media harvested at different time points, including: 0, 10 min, 30 min, 1 h, 3 h and 6 h. S^35^-labeled KAP recombinant protein was immunoprecipitated (IP) with rat mAb against HA epitope (ROCHE) following standard protocols. Conditioned culture media was centrifuged at 5000 rpm for 5 min and concentrated with Amicon® Ultra-4 de 5000 NMWL filters (Millipore). IP proteins and concentrated media were loaded in 15% SDS polyacrylamide gels (SDS-PAGE). After electrophoresis, gels were fix in a solution containing 50% methanol and 10% acetic acid for 30 min, followed for 5 min incubation in 7% methanol, 7% acetic acid and 1% glycerol. Gels were exposed to autoradiographic films.

### Lentiviral vector production and stable KAP expression in HK-2 cells

Viruses were generated in HEK293T cells with four plasmids providing vector, gag-pol and env functions at a ratio of 1:1:3:5 (VSVG:RTR2:PKGPIR:Transfer vector encoding for KAP fused to the HA epitope), as previously described^39^. The viral vector-containing medium was applied to the HK-2 cell line twice. The human proximal tubule epithelial cell line HK-2 (CRL-2190), immortalized by transduction with HPV-16, was obtained from the American Type Culture Collection (ATCC). Seventy-two h post-infection, the medium was removed and cells were incubated with normal growth medium for 5 days until Puromycin (Sigma–Aldrich) at 1 µg/mL was added to culture medium for selection of transduced cells. Cells infected with the empty transfer vector FUW served as a control.

### Western blot analysis

Control and KAP-HA expressing HK-2 cells were washed with PBS and lysed in whole-cell lysis buffer (1% Triton X-100, 20 mM HEPES [pH 7.4], 2 mM EGTA, 1 mM DTT, 50 mM β-glycerophospate, 10% glycerol, 1 mM NaVO_4_) with proteinase inhibitors. Western blot assays were performed as previously described^39^, using rat mAb against HA (ROCHE), rabbit mAb against β-actin (Sigma-Aldrich) and mouse mAb against pSTAT3 Y705 (Cell Signaling) diluted in blocking solution. Horseradish peroxidase-conjugated secondary antibodies were obtained from DAKO (Glostrup, Denmark). Blots were developed with the enhanced chemiluminiscence substrate ECL plus Western blotting detection system (GE Healthcare UK, Buckinghsmshire, UK).

### Statistical Analyses

Statistical analyses were performed using GraphPad Prism version 6.0d (GraphPad Software, USA). Unless otherwise stated, data were analyzed by two-way ANOVA followed by Tukey test.

Regarding microfluidic cards analysis, the selection of differentially expressed elements was performed following the Livak method and implemented with R scripts, using Student’s *t*-test in order to find significant differences in expression levels between groups^40^. Raw data was provided as SDS format files from ABI 7900 PCR System. All of them were loaded and properly re-formated with custom R scripts^41^ developed for this purpose at the VHIR’s Statistics and Bioinformatics Unit (UEB). The steps of the analysis have been defined in three main points: (a) quality control of the samples, focused on outliers detection; (b) data normalization; and (c) selection of differentially expressed features, separately for each specific comparison (named C1, C2, C3 and C4 in Fig. 2). These process have been applied independently for each type of tissue considered in the experiment (Adipose tissue and Liver). Quality control has been conducted with specific R scripts implemented at the UEB, adapting the *HTqPCR Bioconductor’s package* described by Dvinge^42^. No outliers have been detected, and thus no samples have been ruled out after these quality control checking. Data normalization was performed with UEB customized R scripts, based on the standard Livak method known as ΔΔCt computation^40^, using as endogenous controls the genes *Actb*, *Gapdh* and *Ppia* for the liver, and *Ppia* for the adipose. They were the most stable ones, following statistical criteria based on its coefficient of variation.

The selection of differentially expressed elements has been done by the Livak method and implemented with R scripts, using Student’s *t*-test, to find significant differences in expression levels between groups. More specifically, in comparisons C1, C2 and C3, ΔCt values between groups have been used for the test; while ΔΔCt against μ = 0 have been tested in comparison C4. Significance levels have been established at α = 0.05 in all cases.

### Data Availability

All data generated or analyzed during this study are included in this published article (and its Supplementary Information files).

## Electronic supplementary material

Supplementary info file
