# Supplementary material for: Kidney Androgen-Regulated Protein (KAP) Transgenic Mice Are Protected Against High-Fat Diet Induced Metabolic Syndrome
Source: Sci Rep. 2017 Nov 23;7:16102. doi: 10.1038/s41598-017-16487-y (PMC5701062; doi:10.1038/s41598-017-16487-y)
Supplement: Supplementary file 1 — Supplementary info file [file 41598_2017_16487_MOESM1_ESM.pdf]

Kidney Androgen-Regulated Protein (KAP) Transgenic Mice Are Protected Against High-Fat Diet Induced Metabolic Syndrome

Beatriz Bardaji de Quixano [1], Josep A. Villena[2], Miguel Aranda[1], Gemma Brils [1], Antoni Cuevas [1], Théana Hespel[1], Haizea Lekuona[1], Cristina Súarez[1], Olga Tornavaca [1], Anna Meseguer [1, 3, 4, 5]

[1]Fisiopatologia Renal. Centre d'Investigacions en Bioquímica i Biologia Molecular (CIBBIM). Institut de Recerca Vall d'Hebron (VHIR), Barcelona, Spain.

[2]Laboratory of Metabolism and Obesity. Vall d'Hebron Institut de Recerca (VHIR), Barcelona, Spain.

[3]Departament de Bioquímica i Biologia Molecular. Unitat de Bioquímica de Medicina. Universitat Autònoma de Barcelona. Bellaterra (Barcelona) Spain.

[4]Instituto Reina Sofía de Investigación Nefrológica. Fundación Renal Íñigo Álvarez de Toledo. Spain.

[5]Red de Investigación Renal (REDINREN), Instituto Carlos III-FEDER, Madrid, Spain.

Address for correspondence:

Anna Meseguer, Ph.D.

Fisiopatologia Renal. CIBBIM.VHIR.

Pg. Vall d'Hebron 119-129. 08035 Barcelona. SPAIN.

Tel: 34-934894061.

FAX: 34-934894015.

E-mail: ana.meseguer@vhir.org

Short Title:

KAP and metabolic syndrome

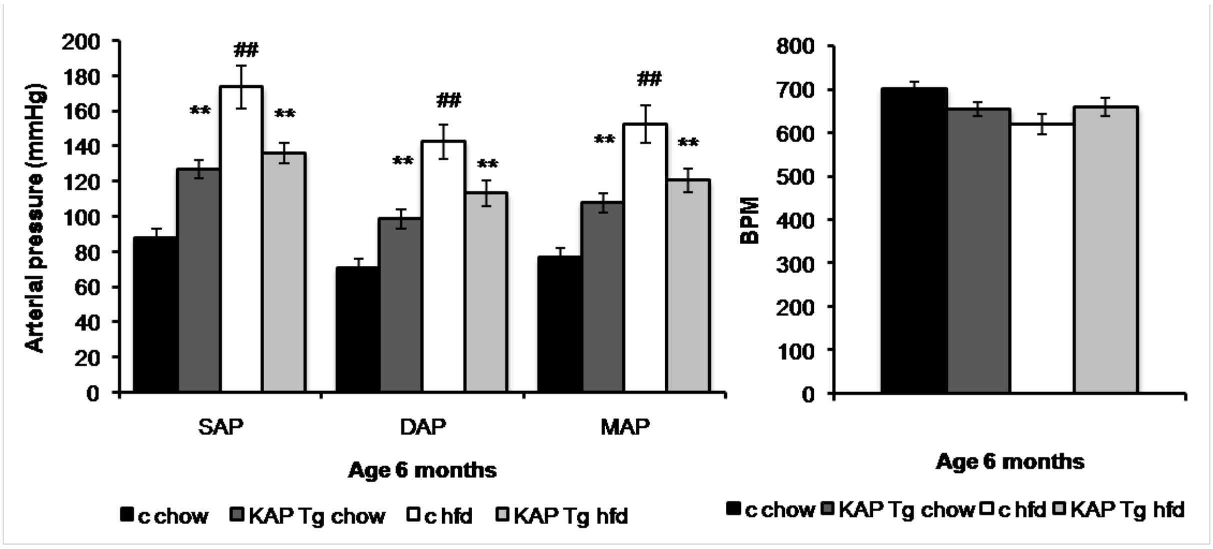

Figure S1. Hemodynamic analyses

Evening tail-cuff measurements of systolic (SAP) diastolic (DAP) and mean arterial pressure (MAP) in Tg and control littermate mice fed on standard control diet (chow) or high fat diet (HFD), at 6-month of age (left panel). Mean and SEM from one experiment (n=8 per group) are represented. Statistical analysis on AUC ** $p<0.01$ control vs Tg, ## $p<0.01$ chow vs HFD. Heart rate measurements in the four groups of mice (right panel). Error bars correspond to SEM.
